# Nanowire Oligomer Waveguide Modes towards Reduced Lasing Threshold

**DOI:** 10.3390/ma13235510

**Published:** 2020-12-03

**Authors:** Henrik Mäntynen, Nicklas Anttu, Harri Lipsanen

**Affiliations:** 1 Department of Electronics and Nanoengineering, Aalto University, P.O. Box 13500, FI-00076 Aalto, Finland; nicklas.anttu@aalto.fi (N.A.); harri.lipsanen@aalto.fi (H.L.); 2 Physics, Faculty of Science and Engineering, Åbo Akademi University, FI-20500 Turku, Finland

**Keywords:** semiconductor nanowire, oligomer, waveguide, guided mode, lasing threshold

## Abstract

Semiconductor nanowires offer a promising route of realizing nanolasers for the next generation of chip-scale optoelectronics and photonics applications. Established fabrication methods can produce vertical semiconductor nanowires which can themselves act both as a gain medium and as a Fabry–Pérot cavity for feedback. The lasing threshold in such nanowire lasers is affected by the modal confinement factor and end facet reflectivities, of which the substrate end reflectivity tends to be limited due to small refractive index contrast between the nanowire and substrate. These modal properties, however, also depend strongly on the modal field profiles. In this work, we use numerical simulations to investigate waveguide modes in vertical nanowire oligomers (that is, arrangements of few vertical nanowires close to each other) and their modal properties compared to single nanowire monomers. We solve for the oligomer waveguide eigenmodes which are understood as arising from interaction of monomer modes and further compute the reflectivity of these modes at the end facets of the nanowires. We consider either the nanowires or an additional coating layer as the gain medium. We show that both types of oligomers can exhibit modes with modal properties leading to reduced lasing threshold and also give directions for further research on the topic.

## 1. Introduction

Semiconductor lasers are vital in modern optoelectronics and photonics, and there is a strong interest in integrating nanolasers in chip-scale systems to realize next generation of applications in optoelectronics and photonics, such as optical interconnects for data transfer [1]. One promising route to realizing integrated nanolasers is by using semiconductor nanowires which can confine and guide optical fields and act as the gain medium [2]. Semiconductor nanowires can be fabricated not only by top-down etching but also by bottom-up growth via either metallic nanoparticles (vapor–liquid–solid growth, VLS [3,4]) or mask-based selectivity (selective-area epitaxy growth, SAE [5,6]). In particular, bottom-up grown group III–V compound semiconductors offer gain media spanning the visible and near-infrared wavelength range while also allowing integration on the technologically important silicon substrates [6].

The nanowire geometry leads to field confinement and waveguiding along the nanowire axis while back-reflections from the end facets result in a natural Fabry–Pérot cavity for feedback. Therefore, the semiconductor nanowire itself realizes a simple laser structure provided that it can be pumped either optically or electrically to reach lasing. The field confinement can actually result in modal gain that is higher than the gain in bulk semiconductor material (under equivalent level of excitation), the ratio of which can be defined as the modal confinement factor [7]. The guided modes and their modal confinement factor and reflectivities in a single nanowire have been quite thoroughly investigated [4,6,8,9]. Vertical growth of III–V semiconductor nanowires on a III–V or a Si substrate is desired for well-defined end facets, but this tends to result in a small refractive index contrast between the nanowire and the substrate. Consequently, obtaining a sufficiently high modal reflectivity at the nanowire–substrate interface to provide a strong enough feedback for lasing can become problematic [6,8], although it is in some cases possible to alter the fabrication process to include a structure that increases the reflection [6]. Nanowires lying horizontally on a substrate of smaller refractive index can avoid this issue [4], but such nanowires cannot be directly grown and any transfer processes significantly complicate the fabrication. However, the modal reflectivity does not simply follow from the refractive index contrast but also depends on the modal field profile [9] which is also associated with the modal confinement factor [7]. It is therefore conceivable that some of the various modes available in vertical nanowire oligomers (arrangements of few vertical nanowires close to each other) could exhibit improved modal reflectivities together with a high modal confinement factor.

The aim of our present work is to use numerical simulations to investigate waveguide modes in vertical nanowire oligomers and their suitability for lasing based on their modal confinement factor and end facet reflectivity properties. We choose to focus on nanowire dimers and tetramers specifically. The oligomer waveguide modes can be understood to arise due to the overlapping and interaction of monomer modes in the individual nanowires, somewhat analogous to the hybridization model for resonant excitations in nanoparticle oligomers [10]. To the best of our knowledge, the modal properties of nanowire oligomer waveguide modes have not been previously reported or their hybridization origin discussed, at least beyond recognizing coupled HE${}_{11}$ modes leading to birefringence in nanowire dimers [11,12]. Here, we will show that InGaAs nanowire dimers and tetramers on a GaAs substrate (assuming bottom-up growth) emitting in the telecommunication C band wavelength range can, indeed, possess modes that exhibit improved modal confinement factor and modal reflectivities compared to the monomer modes from which they originate. Furthermore, we also consider an alternative scheme where Si nanowire dimers and tetramers on a Si substrate (assuming top-down fabrication) provide the waveguiding while the gain is separated to a coating of Er-doped alumina. This high-gain coating material can be fabricated fully conformally via atomic-layer deposition (ALD) and emits in the telecommunication C band wavelength range (specifically the transition at 1533 nm wavelength) [13]. We will show that modes in the coated dimer and tetramer can also obtain better modal properties than the corresponding coated monomer modes. These promising results warrant further research on the topic for which we will indicate directions.

## 2. Simulation Methods

In a nanowire laser, the lasing guided mode propagates along the nanowire axis (taken here to be along the *z*-direction) and is partially reflected back at the end facets. Therefore, the expression for threshold gain (per unit length) with the lasing mode in such a Fabry–Pérot cavity can be written as

(1) $$g_{\mathrm{th}}=\alpha_{r}+\alpha_{p}=\frac{1}{L}\ln\left(\frac{1}{\left|r_{1}\right|\left|r_{2}\right|}\right)+\alpha_{p}\;$$

where $\alpha_{r}$ denotes the end facet reflection losses, $\alpha_{p}$ denotes the optical losses during propagation along the cavity, *L* is the cavity length (assuming that the active region spans the entire cavity length), and $\left|r_{1}\right|$ and $\left|r_{2}\right|$ are the modal field reflection coefficient magnitudes at the ends of the cavity (note that $R={\left|r\right|}^{2}$, where *R* is the modal reflectivity for intensity) [14] (pp. 143–147). Assuming that the optical propagation losses are small compared to reflection losses at the nanowire ends and using the previously mentioned definition of the modal confinement factor (Γ), we can approximate and re-write Equation (1) as:

(2) $$\Gamma g_{\mathrm{th},\mathrm{bulk}}\approx \frac{1}{L}\ln\left(\frac{1}{\left|r_{1}\right|\left|r_{2}\right|}\right)\;.$$

Therefore, we can define a unitless modal cost function

(3) $$f_{\mathrm{mc}}\equiv \frac{1}{\Gamma }\ln\left(\frac{1}{\left|r_{1}\right|\left|r_{2}\right|}\right)\;$$

which should be minimized in order to minimize the required threshold gain. The assumption $\alpha_{r}\gg \alpha_{p}$ is often made for nanowire lasers based on relatively large reflection losses due to typically short cavity length and limited end facet reflectivities (compared to what can be achieved with larger scale mirror structures like distributed Bragg reflector stacks).

**Figure 1:**
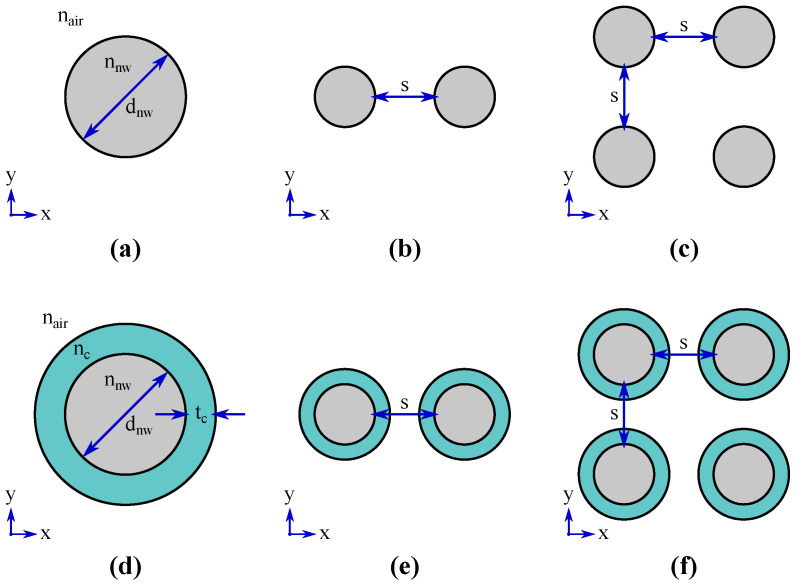
Nanowire oligomer waveguide *xy*-plane cross-sections. (**a**) monomer; (**b**) dimer; (**c**) tetramer; (**d**) coated monomer; (**e**) coated dimer; (**f**) coated tetramer. The nanowire diameter $d_{\mathrm{nw}}$, coating thickness $t_{c}$, nanowire separation *s*, and refractive indices $n_{\mathrm{nw}}$, $n_{c}$, and $n_{\mathrm{air}}=1$ are also indicated.

**Figure 2:**
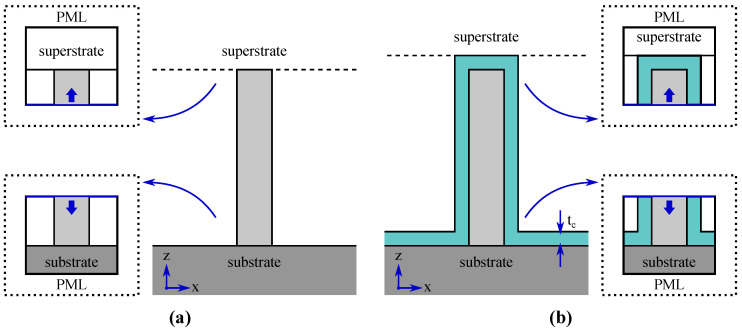
Nanowire monomer waveguide *xz*-plane cross-sections. (**a**) uncoated nanowire; (**b**) coated nanowire. The call-outs show the computational domains (enclosed in perfectly matched layers, PMLs) of the simulations with launching of the guided mode from the input port indicated with arrows. The coating thickness $t_{c}$ is also indicated.

We use the finite element method (COMSOL Multiphysics^®^ 5.5 software with the Wave Optics Module) to solve the nanowire oligomer guided modes, their modal confinement factor in the active region, and reflections from the nanowire ends. First, the waveguide guided modes are solved from 2D horizontal *xy*-plane cross-section models (Mode Analysis), the geometries of which are illustrated in Figure 1. The guided modes are eigenmode solutions with the propagation constant $\beta_{z}$ as the eigenvalue (equivalently, the mode effective refractive index $n_{e}$ can be taken as the eigenvalue since $\beta_{z}=2\pi n_{e}/\lambda_{0}$, where $\lambda_{0}$ is the wavelength in free space). When the fields of a guided mode (denoted here with an index ν) are solved, the modal confinement factor can be computed as [7]

(4) $$\Gamma_{\nu }=\frac{\frac{n_{a}}{2\eta_{0}}\int_{S_{a}}{\left|{\bar{E}}_{\nu }\right|}^{2}dxdy}{\frac{1}{2}\int_{S_{\infty }}ℜ\left\{{\bar{E}}_{\nu }\times {\bar{H}}_{\nu }^{*}\right\}\cdot {\bar{u}}_{z}dxdy}\;$$

where $n_{a}$ is the refractive index (real part) in the active region, $\eta_{0}$ is the characteristic impedance of free space, $S_{a}$ denotes the active cross-section area, $S_{\infty }$ denotes the entire *xy*-plane, ${\bar{E}}_{\nu }$ is the mode electric field, ${\bar{H}}_{\nu }$ is the mode magnetic field, ${}^{*}$ denotes the complex conjugate, and ${\bar{u}}_{z}$ is the unit vector in the *z*-direction. Second, the modal reflection coefficient magnitudes at the substrate and superstrate end of the nanowires ($\left|r_{\mathrm{sub}}\right|$ and $\left|r_{\sup}\right|$) are obtained from 3D models (Wavelength Domain), where the previously solved modal fields are used as input excitation. The vertical *xz*-plane cross-section geometries of the uncoated and coated nanowire monomer are illustrated in Figure 2 with the corresponding computational domains used in simulations indicated. We use the standard overlap integral approach to extract modal power from the 3D field solutions (the specific integral expressions are briefly presented in Appendix A for completeness). Finally, the modal confinement factor and modal reflection coefficient magnitude values are used to calculate the modal cost function value (with Equation (3)).

In order to keep the study focused, we reduce the possible degrees of freedom by focusing on dimers and tetramers and consider them comprising cylindrical nanowires with identical diameters and symmetrical arrangements. The dimer and tetramer oligomers are selected in this study as they clearly demonstrate the guided mode hybridization. There is no fundamental reason why a trimer or a higher-order oligomer beyond the tetramer could potentially not exhibit similar or even better modal properties, although our preliminary results for the trimer indicated no great difference in the achievable modal confinement factor values (not shown here). Furthermore, the circular cross-section allows us to verify the validity of the monomer numerical eigenmode solutions against semi-analytical solutions. The uncoated nanowire monomer corresponds to a two-layer step-index optical fiber for which the semi-analytical mode eigenvalue equations can be found in standard optical fiber textbooks, while the coated nanowire monomer corresponds to a three-layer step-index optical fiber for which the semi-analytical mode eigenvalue equations can be found, e.g., in Refs. [15,16]. We also keep the study focused by not including in the models any surface passivation layers or taking into consideration surface depletion regions and simply take the entire nanowire cross-section as the active region with the uncoated nanowires (note, however, that such extensions for the model are in principle straightforward to implement). The bottom-up growth of III–V nanowires via SAE or VLS actually tend to yield hexagonal cross-section instead of circular [4,5]. However, the difference in effective refractive index and end facet reflections of low-order modes between hexagonal and circular cross-section nanowires have been studied in detail and found to be minimal [9]. Note that, with top-down fabrication, it is in principle possible to obtain nanowires with cross-sections of lower symmetry, including elliptical ones with modes providing form birefringence [17].

We select the materials in the models as follows: the uncoated nanowires are InGaAs on top of a GaAs substrate and the coated nanowires are Si on top of a Si substrate with Er-doped alumina as the coating. In practice, such uncoated nanowires could be fabricated via bottom-up SAE while the coated nanowires could be fabricated via top-down etching with the coating deposited with ALD. We consider a GaAs substrate instead of Si for the uncoated nanowires as this material combination has currently more developed growth processes and hence would allow for easier fabrication of potential proof-of-concept samples. Similarly, top-down etching of Si nanowires allows for using a (100) plane substrate more suited for electronics integration while conventional bottom-up grown GaAs nanowires could be alternatively employed if a (111) plane substrate is used. In any case, no significant difference in the results would be expected due to the relatively small difference in the GaAs and Si refractive indices. At the wavelength 1533 nm, we use the refractive index value n=3.4523 for InAs [18], n=3.3720 for GaAs [19], n=3.4771 for Si [20], and n=1.6500 for Er-doped alumina [13]. For the uncoated In${}_{x}$Ga${}_{1-x}$As nanowires, direct band gap room-temperature emission at the 1533 nm wavelength is expected with the composition x=0.4633, based on calculations with the values reported in [21]. Using linear interpolation between the InAs and GaAs refractive index values to x=0.4633 then yields n=3.4092 for InGaAs. We assume that the imaginary part of the refractive index (related to absorption and gain) is small compared to the real part such that we can model the waveguiding under the approximation $n\approx ℜ\left\{n\right\}$.

The 2D cross-section COMSOL models additionally require a choice for the finite computational domain, boundary conditions, meshing, and solver. We choose a square computational domain and select the width such that the modal fields decay enough before reaching the boundaries. We verify this decay by checking convergence of the eigenvalues with the computational domain width and by inspection of field norm plots. Since the fields are sufficiently decayed, the exact choice for the boundary conditions does not cause significant effects and we can simply select them as perfect electric conductors (PEC). For meshing, we use free triangular mesh for the entire geometry with the “Fine” preset settings, except for the maximum mesh element size, which is varied to assess convergence in the results. We choose the finest value for the maximum mesh element size in each region as $\lambda_{0}/\left(12n\right)$, where $\lambda_{0}$ is the free space wavelength and *n* is the refractive index in the region. The finest values are then scaled with a common factor (≥1) to vary the meshing. We also confirm that the meshing is not limited by the other settings by looking at the change in the system degrees of freedom as the maximum mesh element size is varied. The mode effective refractive indices are obtained with the Mode Analysis at $\lambda_{0}=1533$ nm and the MUMPS solver with the default settings.

The 3D modal reflection COMSOL models handle the substrate and superstrate ends separately (as indicated by the call-outs in Figure 2). The selected guided mode is launched from an input port at the bottom of the computational domain towards the nanowire end, where it will couple to the reflected guided mode, possibly other guided modes (if allowed by the symmetry set by the waveguide geometry and the launched guided mode), leaky modes, and scattered radiation modes. The computational domain is enclosed with PMLs and the port is set as domain backed, so that all fields propagating out of the domain are absorbed in the PMLs without further reflections. The forward propagating and reflected power in the selected guided mode are extracted with Equations (A12)–(A14), for which the modal fields are obtained from the port and the total field can, in principle, be evaluated at any *xy*-plane cross-section along the waveguide. In practice, the extracted power may slightly vary as a function of the position due to numerical errors. Therefore, both the forward propagating and the reflected power are evaluated at several *xy*-planes along the waveguide and the final result is averaged over these. The modal reflection coefficient magnitude is then simply obtained as the square root of the ratio of the reflected power to the forward propagating power.

The reflection models are solved in two steps: first a Boundary Mode Analysis solution for the port and then a Wavelength Domain solution for the full geometry (the MUMPS solver with the default settings is used throughout). The port geometry corresponds to the 2D waveguide cross-section models and the same meshing is applied. The Boundary Mode Analysis is given the selected known guided mode solution as the starting point. The only difference between a 2D cross-section model and the corresponding port in a 3D model is that the boundaries are now PML instead of PEC. However, as already mentioned, as long as the fields decay enough before reaching the boundaries, this difference is not significant for the solving and launching of the mode. Therefore, the Boundary Mode Analysis yields essentially the same solution. The power in the launched guided mode is defined by the port settings, and this value is hence used to verify the power extraction calculations. The full geometry beyond the port is meshed using free tetrahedral mesh, except for the PMLs for which a swept mesh is used (with six elements across). We use the same meshing settings as with the 2D models, but, due to computational budget limitations, convergence of the results is checked by varying the maximum mesh element sizes by a factor of ≥2 (i.e., the mesh is always coarser than with the previously defined finest value). The waveguide length, substrate or superstrate region height, and PML layer thickness and stretching do not affect the physics in the models and are selected purely based on avoiding causing numerical errors or artifacts.

We implement the power extraction calculations in MATLAB^®^ R2019b software for convenience, instead of setting up computations inside the graphical user interface of COMSOL combining both the Boundary Mode Analysis and the Wavelength Domain results. We perform the numerical integration by using the 16-point quadrature rule on triangles of Ref. [22]. First, the port triangular mesh is exported from COMSOL and the corresponding quadrature points are determined. The port guided mode fields and the total field on several *xy*-planes are then interpolated to these points in COMSOL, and the results are exported to MATLAB for performing the integration using the field values at the points and the corresponding quadrature weights and triangle areas. Although using the port mesh is perfectly valid for any *xy*-plane cross-section along the entire waveguide region, the interpolation does not necessarily make the best use of the shape functions COMSOL uses for the field solutions since the mesh at the *xy*-planes beyond the port is actually tetrahedral. Regardless, this method is accurate enough as verified by the good agreement of the computed forward propagating power to the input power set for the port.

## 3. Results

We investigate properties of the nanowire oligomer modes as a function of the geometry by varying diameter of the nanowires, coating thickness, and separation of the nanowires. With the uncoated nanowires, the diameter is varied from 300 nm to 500 nm with the 5 nm step, and the separation is varied from 10 nm to 400 nm with non-uniform stepping. The waveguide modes are expected to be more sensitive to the nanowire separation at small values, and the step size is hence selected as follows: 5 nm step between 10 nm and 50 nm separation, 10 nm step between 50 nm and 100 nm, 20 nm between 100 nm and 200 nm separation, and 40 nm between 200 nm and 400 nm separation. The chosen step sizes are expected to provide sufficient resolution while also being comparable to or below feasible fabrication tolerances. The chosen diameter range is suitable for fabrication via either bottom-up or top-down methods and the larger diameters support also TE${}_{01}$ and TM${}_{01}$ modes in the nanowires in addition to the fundamental HE${}_{11}$ mode. For any higher order modes, the diameter remains below their respective cut-offs (with the considered wavelength and refractive indices). These modes tend to get more confined inside the nanowire with increasing diameter leading to smaller overlap and weaker interaction (hybridization) with modes in neighboring nanowires. Although difficult to achieve fabrication-wise, the smallest separation is selected to ensure strong hybridization of modes in neighboring nanowires while the largest separation is expected to show much weaker hybridization effect. For practical device fabrication, separations larger than around 50 nm might be more feasible. With further increase of the separation, the modes in each nanowire would eventually become independent.

With the coated nanowires, the diameter is varied from 200 nm to 300 nm with 20 nm step, the coating thickness is varied from 200 to 400 nm with 20 nm step, and the separation is varied from 10 nm to 400 nm with the same stepping as with the uncoated nanowires. The chosen coating thickness step size is expected to provide sufficient resolution (fabrication via ALD is actually capable of much greater accuracy). The coating is chosen to be relatively thick compared to the diameter in order to promote field confinement in the coating, while still keeping the overall extent not much larger than with the uncoated nanowires. Although ALD is more suited for thinner coatings with precise thickness control, the largest thickness of 400 nm considered here is still feasible (for example, in Ref. [13], the deposited Er-doped alumina coating was 150 nm thick). However, thinner coatings might be preferable in practical applications. Furthermore, the coating thickness and nanowire separation are also selected such that the coatings are always overlapping or at least touching, since we want to avoid a situation where the fields would focus in an air gap forming between the coated wires. In this parameter range, although the mode behavior is more complicated than with the uncoated nanowires, still only HE${}_{11}$, TE${}_{01}$, and TM${}_{01}$ modes can propagate.

The different modes in the nanowire oligomers can be understood as having hybridized from monomer modes in each nanowire, and these combinations can be deduced based on symmetry considerations. In particular, HE${}_{11}$ mode has a clear direction in both its transverse electric and transverse magnetic field, and, due to the rotational symmetry of the monomer geometry, it can be oriented in any angle in the cross-section plane. Therefore, HE${}_{11}$ mode in a nanowire monomer is somewhat analogous to a resonant dipole excitation in a nanoparticle. Nanoparticle dimer and tetramer geometries have the symmetry point group D${}_{2h}$ and D${}_{4h}$, respectively, yielding (in-plane) dipole-excitation-based symmetry-adapted eigenmodes as follows: four non-degenerate hybridized modes in the dimer and two doubly-degenerate and four non-degenerate hybridized modes in the tetramer [10]. Indeed, we observe similar HE${}_{11}$-mode-based hybridized waveguide modes in our nanowire dimers and tetramers. These hybridized modes exhibit the strengthened or weakened field, in the region between the nanowires, where the overlapping single-nanowire mode contributions are parallel or anti-parallel, respectively (note, however, that the hybridized modes are not just a sum of the single-nanowire modes but contain the effect of interaction as well). The analogy with the nanoparticle oligomer resonant eigenmode excitations is not quite complete as the hybridized waveguide modes have a continuous dispersion in $\beta_{z}$ and hence do not, strictly speaking, follow a classification to bonding (lower energy) and antibonding (higher energy) hybridized states. Due to the rotational symmetry of TE${}_{01}$ and TM${}_{01}$ modes, only two hybridized modes for each (with field orientation corresponding to constructive and destructive interaction) exist for the dimer. With the tetramer, however, more combinations (some of which are degenerate) are possible. The same mode hybridization is observed also in the coated nanowires.

Due to the large number of hybridized modes, especially with the tetramer, we choose to consider only a few select ones in our analysis. For the uncoated nanowire dimer and tetramer, we select the HE${}_{11}$- and TE${}_{01}$- or TM${}_{01}$-based hybridized modes that reach the highest modal confinement factors. With the coated nanowire oligomers, however, the TE${}_{01}$- or TM${}_{01}$-based hybridized modes tend to reach modal confinements comparable with the HE${}_{11}$-based hybridized modes only at the largest coating thicknesses and separations. Therefore, for the coated nanowire dimer and tetramer, we consider only the HE${}_{11}$-based hybridized modes reaching the highest modal confinement factor. Additionally, we will compare these modes to their oppositely oriented configuration counterparts. Here, and in the following, the modal confinement factor refers to the active region, which is the nanowire volume for the uncoated oligomers and the coating volume for the coated oligomers.

### 3.1. Uncoated Nanowire Oligomers

**Figure 3:**
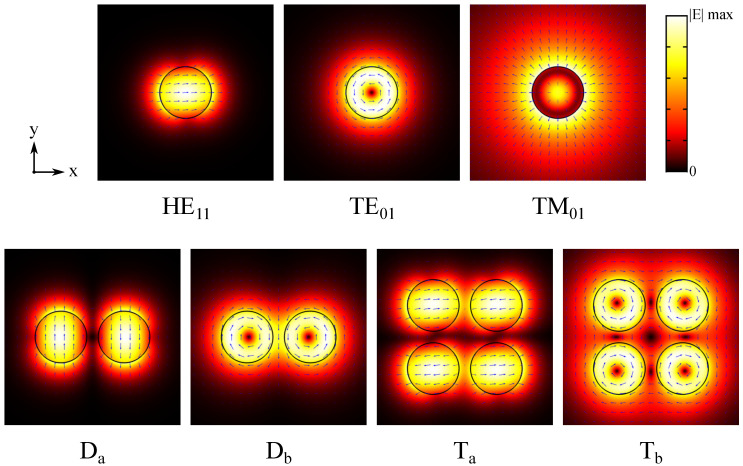
Electric field plots in the *xy*-cross-section of the uncoated nanowire oligomer waveguide modes included in the analysis. The color map shows the field norm $\left|\bar{E}\right|$ and the blue arrows indicate the transverse field direction. Here, the nanowire diameter is 450 nm and nanowire separation is 100 nm.

The selected uncoated nanowire oligomer modes are shown in Figure 3 (including the monomer modes for reference). We denote the two investigated dimer modes as D${}_{a}$ and D${}_{b}$ and the two investigated tetramer modes as T${}_{a}$ and T${}_{b}$, as indicated in the figure. In the dimer mode D${}_{a}$, transverse electric fields of the HE${}_{11}$ modes in the nanowires are oriented along the *y*-axis and in opposite directions resulting in a weaker field between the nanowires. In the dimer mode D${}_{b}$, on the other hand, electric fields of the TE${}_{01}$ modes in the nanowires have opposite handedness and are hence oriented in the same direction in the gap between the nanowires resulting in a stronger field. The tetramer modes T${}_{a}$ and T${}_{b}$ are also hybridized from HE${}_{11}$ and TE${}_{01}$ modes, respectively, but both in configurations leading to destructive field interaction. In the tetramer mode T${}_{a}$, transverse electric fields of the HE${}_{11}$ modes in the nanowires are oriented along the *x*-axis such that the overlapping fields of nearest-neighbor nanowires are in the opposite direction. In the tetramer mode T${}_{b}$, the TE${}_{01}$ modes in the nanowires have the same handedness and hence the electric fields overlapping at the gaps between the nanowires are in opposite directions. Of all the observed dimer and tetramer modes in the studied parameter space, D${}_{a}$ and T${}_{a}$ reached the overall highest modal confinement factor, respectively, while D${}_{b}$ and T${}_{b}$ reached the highest modal confinement factor of non-HE${}_{11}$-based modes, respectively (these results are not shown here).

The minimum modal cost function values in the studied parameter space obtained from the simulations with the selected uncoated nanowire oligomer modes are given in Table 1. The table also lists the corresponding modal confinement factors, substrate, and superstrate modal reflection coefficient magnitudes, and the diameters and separations of the nanowires. In order to provide further information on the effect of the geometry on the results, we show in the following the sensitivity of the modal cost function value, modal confinement factor, and modal reflection coefficient magnitudes at the optimal point of each mode to variation in either the diameter or separation of the nanowires.

**Figure 4:**
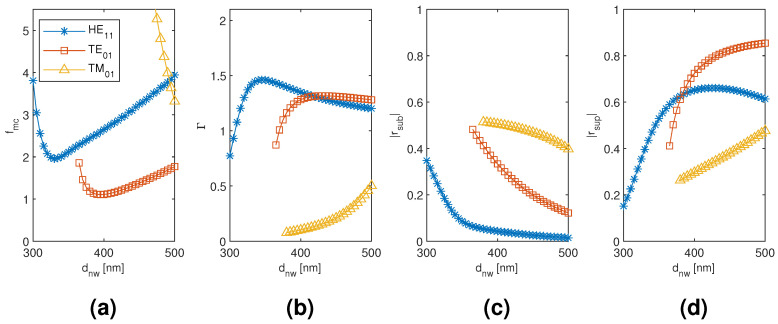
The results for the nanowire monomer HE${}_{11}$, TE${}_{01}$, and TM${}_{01}$ mode as a function of the nanowire diameter. (**a**) modal cost function value; (**b**) modal confinement factor; (**c**) substrate modal reflection coefficient magnitude; (**d**) superstrate modal reflection coefficient magnitude.

The results for the monomer HE${}_{11}$, TE${}_{01}$, and TM${}_{01}$ mode are plotted in Figure 4 as a function of the nanowire diameter. The smallest modal cost function value is reached with the TE${}_{01}$ mode with a large margin to the HE${}_{11}$ and TM${}_{01}$ mode values. Although the HE${}_{11}$ mode is able to reach higher modal confinement factor, the modal reflection coefficient magnitudes are significantly smaller than with the TE${}_{01}$ mode. With larger nanowire diameter, the modal fields tend to focus more inside the nanowire, and, since there is very small refractive index contrast between the substrate and the nanowire, the substrate end modal reflection coefficient magnitude tends to get smaller with all the modes. Conversely, there is a significant refractive index contrast between the nanowire and air, and the superstrate end modal reflection coefficient magnitudes hence show the opposite trend with the nanowire diameter. These opposite trends present the main trade-off in obtaining the minimum modal cost function value, except with the TM${}_{01}$ mode for which the main limiting factor is the low modal confinement factor even at the largest nanowire diameter considered. Our monomer results seem to be in line with other previously reported single nanowire results [4,6,8] and form a point of comparison for the dimer and tetramer results.

**Figure 5:**
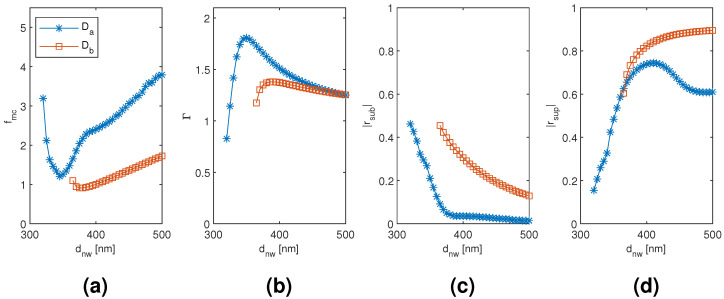
The results for the nanowire dimer D${}_{a}$ and D${}_{b}$ mode as a function of the diameter of the nanowires with the separation of the nanowires corresponding to the point of modal cost function minimum for each mode (as listed in Table 1). (**a**) modal cost function value; (**b**) modal confinement factor; (**c**) substrate modal reflection coefficient magnitude; (**d**) superstrate modal reflection coefficient magnitude.

**Figure 6:**
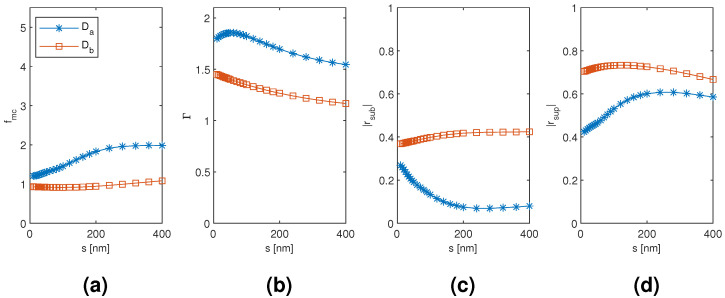
The results for the nanowire dimer D${}_{a}$ and D${}_{b}$ mode as a function of the separation of the nanowires with the diameter of the nanowires corresponding to the point of modal cost function minimum for each mode (as listed in Table 1). (**a**) modal cost function value; (**b**) modal confinement factor; (**c**) substrate modal reflection coefficient magnitude; (**d**) superstrate modal reflection coefficient magnitude.

The results for the dimer D${}_{a}$ and D${}_{b}$ mode are plotted in Figure 5 and Figure 6 as a function of the diameter and separation of the nanowires, respectively, with the other dimension corresponding to the point of modal cost function minimum for each mode (as listed in Table 1). The diameter dependence with the D${}_{a}$ and D${}_{b}$ mode results is qualitatively very similar to the HE${}_{11}$ and TE${}_{01}$ mode of the monomer, respectively, including the substrate–superstrate reflection trade-off. However, at the modal cost function minimum and overall, the D${}_{a}$ mode is able to reach larger modal confinement factor and modal reflection coefficient magnitudes than the HE${}_{11}$ mode. With the D${}_{b}$ mode, the modal confinement factor at smaller diameters is improved, and there is a small increase in the modal reflection coefficient magnitudes compared to the TE${}_{01}$ mode. Consequently, the two hybridized dimer modes show considerable improvement in the minimum modal cost function value compared to the corresponding monomer modes (down from 1.956 to 1.208 and from 1.105 to 0.913 with the D${}_{a}$ and D${}_{b}$ mode, respectively). The D${}_{a}$ mode results are also sensitive to the nanowire separation, although to a lesser extent than to the diameter. The minimum modal cost function value is obtained at the smallest considered separation and the value increases with increasing separation. This seems to be mainly due to the relatively large drop in the substrate end modal reflection coefficient magnitude with increasing separation. However, the change is rather gradual and the modal cost function value is still approximately 1.455 at 100 nm separation. The D${}_{b}$ mode modal cost function value, on the other hand, is highly insensitive to varying the separation from the optimal point as the changes in modal confinement factor and reflection coefficient magnitudes essentially seem to cancel each other out.

**Figure 7:**
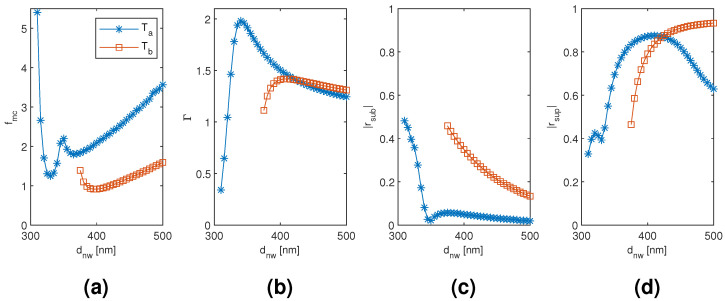
The results for the tetramer T${}_{a}$ and T${}_{b}$ mode as a function of the diameter of the nanowires with the separation of the nanowires corresponding to the point of modal cost function minimum for each mode (as listed in Table 1). (**a**) modal cost function value; (**b**) modal confinement factor; (**c**) substrate modal reflection coefficient magnitude; (**d**) superstrate modal reflection coefficient magnitude.

**Figure 8:**
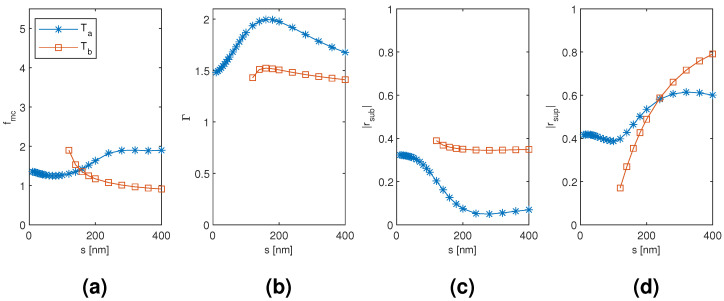
The results for the tetramer T${}_{a}$ and T${}_{b}$ mode as a function of the separation of the nanowires with the diameter of the nanowires corresponding to the point of modal cost function minimum for each mode (as listed in Table 1). (**a**) modal cost function value; (**b**) modal confinement factor; (**c**) substrate modal reflection coefficient magnitude; (**d**) superstrate modal reflection coefficient magnitude.

The results for the tetramer T${}_{a}$ and T${}_{b}$ mode are plotted in Figure 7 and Figure 8 as a function of the diameter and separation of the nanowires, respectively, with the other dimension corresponding to the point of modal cost function minimum for each mode (as listed in Table 1). The modal cost function minimum and corresponding modal confinement factor, modal reflection coefficient magnitudes, and nanowire diameter with the T${}_{a}$ and T${}_{b}$ mode are quite close to those of the D${}_{a}$ and D${}_{b}$ mode of the dimer, respectively. Furthermore, the nanowire diameter dependence is qualitatively similar. However, notable differences arise in the nanowire separation dependence. First, the separation corresponding to the modal cost function minimum is 80 nm with the T${}_{a}$ mode compared to 10 nm with the D${}_{a}$ mode and 400 nm with the T${}_{b}$ mode compared to 100 nm with the D${}_{b}$ mode. With the T${}_{a}$ mode, this seems to be mainly due to the modal confinement factor peaking at a separation of around 160 nm instead of around 50 nm as with the D${}_{a}$ mode. With the T${}_{b}$ mode, the modal confinement factor and substrate end modal reflection coefficient magnitude seem even more insensitive to the nanowire separation than with the D${}_{b}$ mode. However, the superstrate end modal reflection coefficient magnitude is instead highly sensitive to the separation and varies from 0.170 at 120 nm separation to 0.791 at 400 nm. Consequently, the dependence on separation in the superstrate end modal reflection coefficient magnitude dominates in the T${}_{b}$ mode modal cost function value, and the minimum goes to 400 nm, i.e., the largest separation considered.

### 3.2. Coated Nanowire Oligomers

**Figure 9:**
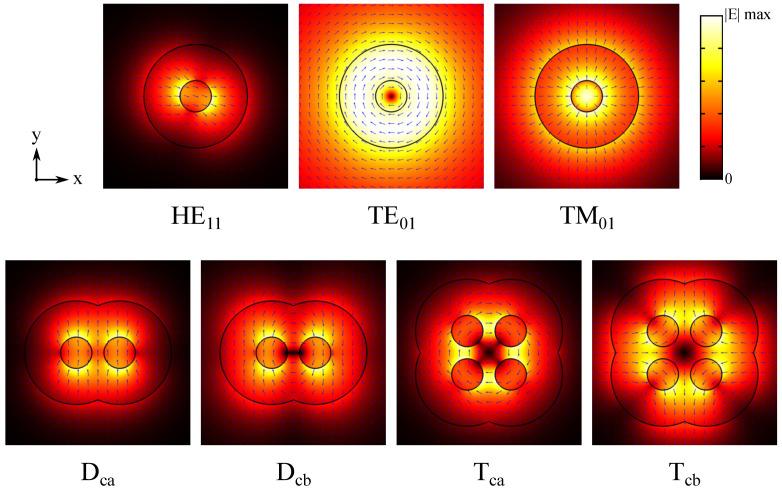
Electric field plots in the *xy*-cross-section of the coated nanowire oligomer waveguide modes included in the analysis. The color map shows the field norm $\left|\bar{E}\right|$ and the blue arrows indicate the transverse field direction. Here, the nanowire diameter is 260 nm, coating thickness is 300 nm, and nanowire separation is 100 nm.

The selected coated nanowire oligomer modes are shown in Figure 9 (including the monomer modes for reference). We denote the two investigated coated dimer modes as D${}_{\mathrm{ca}}$ and D${}_{\mathrm{cb}}$ and the two investigated coated tetramer modes as T${}_{\mathrm{ca}}$ and T${}_{\mathrm{cb}}$, as indicated in the figure. In the dimer D${}_{\mathrm{ca}}$ mode, transverse electric fields of the HE${}_{11}$ modes in the nanowires are oriented along the *y*-axis and in the same direction resulting in a stronger field between the nanowires. The dimer D${}_{\mathrm{cb}}$ mode is simply the opposing configuration with a weaker field between the nanowires. The tetramer modes T${}_{\mathrm{ca}}$ and T${}_{\mathrm{cb}}$, on the other hand, are different in the sense that the overlapping fields of the diagonally oriented HE${}_{11}$ modes between neighboring nanowires are neither parallel nor anti-parallel but rather crossed. However, we still consider these two modes as complementary as one can be obtained from the other by flipping the HE${}_{11}$ field directions in two diagonal nanowires. Of all the observed coated dimer and tetramer modes in the studied parameter space, the D${}_{\mathrm{cb}}$ and T${}_{\mathrm{cb}}$ mode reached the overall highest modal confinement factor, respectively (these results are not shown here). Another interesting notion is that the D${}_{\mathrm{cb}}$ and T${}_{\mathrm{ca}}$ modes have TE-like transverse field distributions, although the *z*-components are still significant (not shown).

The minimum modal cost function values in the studied parameter space obtained from the simulations with the selected coated nanowire oligomer modes are given in Table 2. The table also lists the corresponding modal confinement factors, substrate, and superstrate modal reflection coefficient magnitudes, diameters of the nanowires, coating thicknesses, and separations of the nanowires. In order to provide further information on the effect of the geometry on the results, we show in the following the sensitivity of the modal cost function value, modal confinement factor, and modal reflection coefficient magnitudes at the optimal point of each mode to variation in the diameter, coating thickness, or separation.

**Figure 10:**
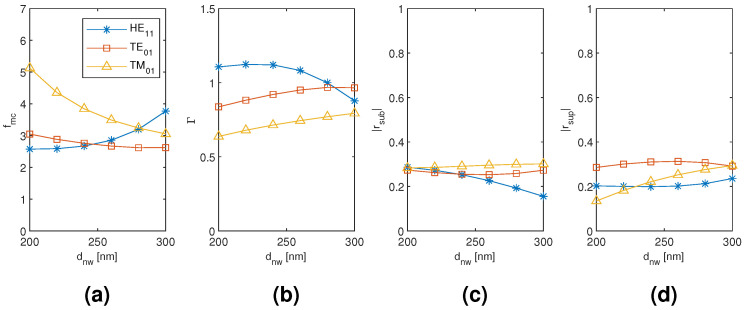
The results for the coated monomer HE${}_{11}$, TE${}_{01}$, and TM${}_{01}$ mode as a function of the nanowire diameter with the coating thickness corresponding to the point of modal cost function minimum for each mode (as listed in Table 2). (**a**) modal cost function value; (**b**) modal confinement factor; (**c**) substrate modal reflection coefficient magnitude; (**d**) superstrate modal reflection coefficient magnitude.

**Figure 11:**
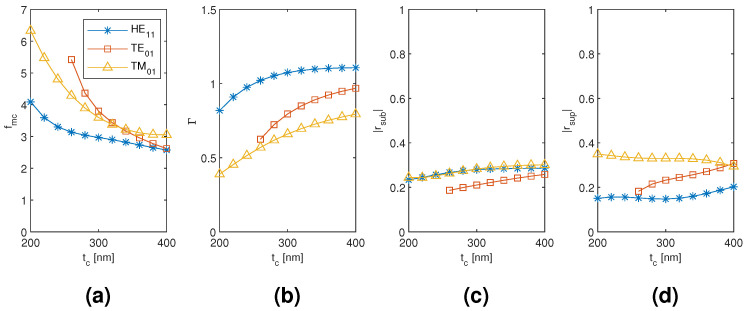
The results for the coated monomer HE${}_{11}$, TE${}_{01}$, and TM${}_{01}$ mode as a function of the coating thickness with the nanowire diameter corresponding to the point of modal cost function minimum for each mode (as listed in Table 2). (**a**) modal cost function value; (**b**) modal confinement factor; (**c**) substrate modal reflection coefficient magnitude; (**d**) superstrate modal reflection coefficient magnitude.

The results for the coated monomer HE${}_{11}$, TE${}_{01}$, and TM${}_{01}$ mode are plotted in Figure 10 and Figure 11 as a function of the nanowire diameter and the coating thickness, respectively, with the other dimension corresponding to the point of modal cost function minimum for each mode (as listed in Table 2). Compared to the uncoated monomer, there is clearly a smaller difference between the results of the modes here, and the modal cost function minima are worse with the HE${}_{11}$ and TE${}_{01}$ mode and slightly better with the TM${}_{01}$ mode. Furthermore, all modes require large coating thickness to improve the modal confinement factor, and still only the HE${}_{11}$ mode reaches values larger than unity. On the other hand, as expected due to the refractive index contrast between the coating and the substrate, the substrate end modal reflection coefficient magnitudes are much less sensitive to the geometry than with the uncoated nanowires. There is also no significant trade-off between the substrate and superstrate end reflection. However, the reflection coefficient magnitudes are overall smaller than with the uncoated monomer, especially for the superstrate. It is not surprising that the superstrate end modal reflection coefficient magnitudes are so much smaller as the refractive index contrast between the coating and air is also significantly smaller than the contrast between nanowire and air with the uncoated nanowire. The HE${}_{11}$ mode has the smallest modal cost function value of 2.574, and it serves as a point of comparison for the coated dimer and tetramer results (the hybridized modes there being HE${}_{11}$-based).

**Figure 12:**
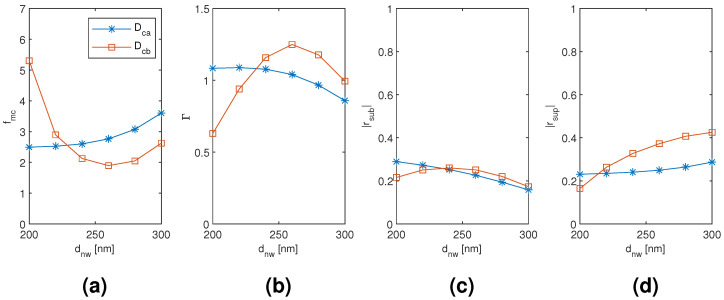
The results for the coated dimer D${}_{\mathrm{ca}}$ and D${}_{\mathrm{cb}}$ mode as a function of the diameter of the nanowires with the coating thickness and the separation of the nanowires corresponding to the point of modal cost function minimum for each mode (as listed in Table 2). (**a**) modal cost function value; (**b**) modal confinement factor; (**c**) substrate modal reflection coefficient magnitude; (**d**) superstrate modal reflection coefficient magnitude.

**Figure 13:**
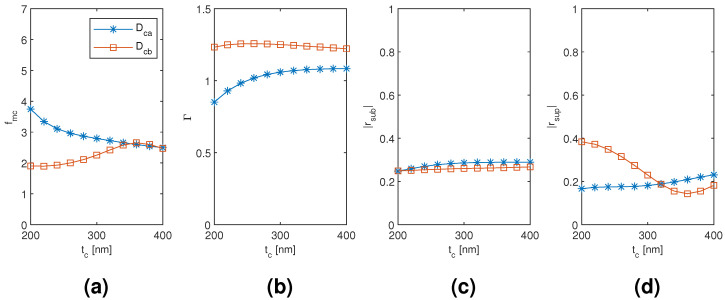
The results for the coated dimer D${}_{\mathrm{ca}}$ and D${}_{\mathrm{cb}}$ mode as a function of the coating thickness with the diameter and separation of the nanowires corresponding to the point of modal cost function minimum for each mode (as listed in Table 2). (**a**) modal cost function value; (**b**) modal confinement factor; (**c**) substrate modal reflection coefficient magnitude; (**d**) superstrate modal reflection coefficient magnitude.

**Figure 14:**
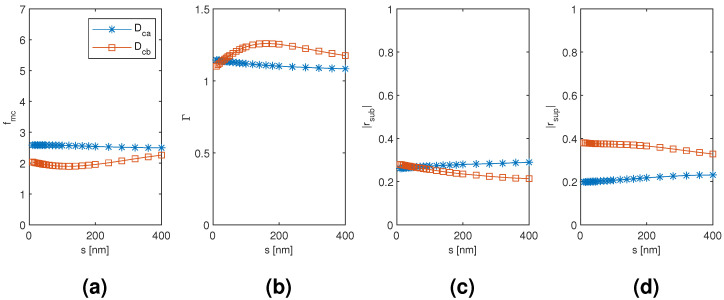
The results for the coated dimer D${}_{\mathrm{ca}}$ and D${}_{\mathrm{cb}}$ mode as a function of the separation of the nanowires with the diameter of the nanowires and the coating thickness corresponding to the point of modal cost function minimum for each mode (as listed in Table 2). (**a**) modal cost function value; (**b**) modal confinement factor; (**c**) substrate modal reflection coefficient magnitude; (**d**) superstrate modal reflection coefficient magnitude.

The results for the coated dimer D${}_{\mathrm{ca}}$ and D${}_{\mathrm{cb}}$ mode are plotted in Figure 12, Figure 13 and Figure 14 as a function of the diameter of the nanowires, the coating thickness, and the separation of the nanowires, respectively, with the two other dimensions corresponding to the point of modal cost function minimum for each mode (as listed in Table 2). The D${}_{\mathrm{ca}}$ mode results seem to follow very closely the coated monomer HE${}_{11}$ mode results with relative insensitivity to the nanowire separation as well, which suggests a weak hybridization effect. The D${}_{\mathrm{cb}}$ mode results, on the contrary, show a noticeable difference in the nanowire diameter and coating thickness dependence of the modal confinement factor and superstrate end modal reflection coefficient magnitude (less so with the substrate end), and the D${}_{\mathrm{cb}}$ mode also shows somewhat greater sensitivity to the nanowire separation than the D${}_{\mathrm{ca}}$ mode. Higher modal confinement factor and superstrate end reflection coefficient magnitude with the D${}_{\mathrm{cb}}$ mode lead to smaller minimum modal cost function value than with the D${}_{\mathrm{ca}}$ mode (1.896 compared to 2.496). Furthermore, the minimum occurs at smaller coating thickness and nanowire separation (220 nm compared to 400 nm and 120 nm compared to 400 nm, respectively).

**Figure 15:**
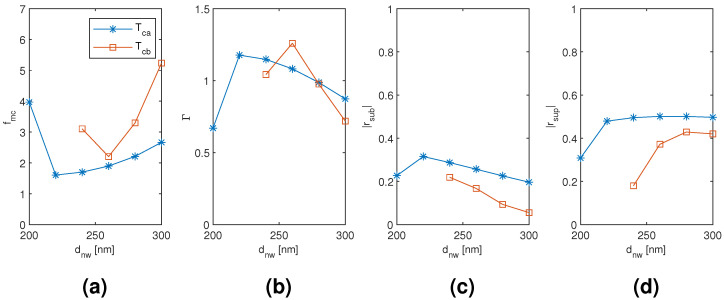
The results for the coated tetramer T${}_{\mathrm{ca}}$ and T${}_{\mathrm{cb}}$ mode as a function of the diameter of the nanowires with the coating thickness and the separation of the nanowires corresponding to the point of modal cost function minimum for each mode (as listed in Table 2). (**a**) modal cost function value; (**b**) modal confinement factor; (**c**) substrate modal reflection coefficient magnitude; (**d**) superstrate modal reflection coefficient magnitude.

**Figure 16:**
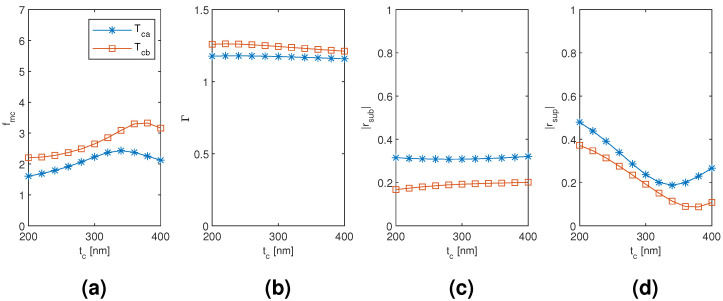
The results for the coated tetramer T${}_{\mathrm{ca}}$ and T${}_{\mathrm{cb}}$ mode as a function of the coating thickness with the diameter and separation of the nanowires corresponding to the point of modal cost function minimum for each mode (as listed in Table 2). (**a**) modal cost function value; (**b**) modal confinement factor; (**c**) substrate modal reflection coefficient magnitude; (**d**) superstrate modal reflection coefficient magnitude.

**Figure 17:**
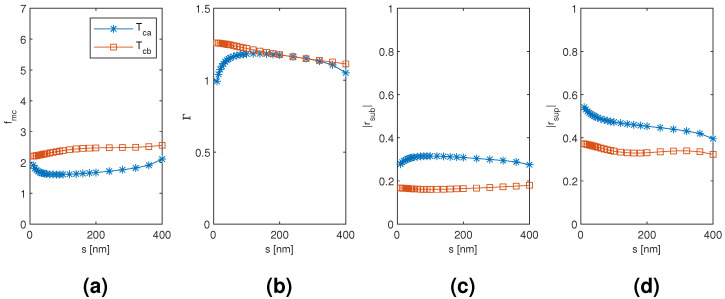
The results for the coated tetramer T${}_{\mathrm{ca}}$ and T${}_{\mathrm{cb}}$ mode as a function of the separation of the nanowires with the diameter of the nanowires and the coating thickness corresponding to the point of modal cost function minimum for each mode (as listed in Table 2). (**a**) modal cost function value; (**b**) modal confinement factor; (**c**) substrate modal reflection coefficient magnitude; (**d**) superstrate modal reflection coefficient magnitude.

The results for the coated tetramer T${}_{\mathrm{ca}}$ and T${}_{\mathrm{cb}}$ mode are plotted in Figure 15, Figure 16 and Figure 17 as a function of the diameter of the nanowires, the coating thickness, and the separation of the nanowires, respectively, with the two other dimensions corresponding to the point of modal cost function minimum for each mode (as listed in Table 2). Both the T${}_{\mathrm{ca}}$ and T${}_{\mathrm{cb}}$ mode reach considerably smaller modal cost function values than the coated monomer HE${}_{11}$ mode (1.607 and 2.204, respectively, compared to 2.574) and at the smallest considered coating thickness instead of the largest (200 nm compared to 400 nm). The improvement seems to be mainly due to increased superstrate end modal reflection coefficient magnitude and also due to increased modal confinement factor. The T${}_{\mathrm{ca}}$ mode reaches the smallest minimum modal cost function value due to larger modal reflection coefficient magnitude at both the substrate and superstrate end than the T${}_{\mathrm{cb}}$ mode, although reaching a smaller maximum modal confinement factor. It is quite interesting that, with both the coated dimer and tetramer, the mode with TE${}_{01}$-like transverse fields (D${}_{\mathrm{cb}}$ and T${}_{\mathrm{ca}}$ mode, respectively) would reach the smaller modal cost function value and with significant margin to the other modes investigated.

## 4. Discussion

Overall, the results indicate that it is possible to obtain improved modal cost function values with either dimer or tetramer hybridized waveguide modes compared to the corresponding monomer modes, applying to both the uncoated and coated nanowire case. In other words, with optimized dimer and tetramer geometries, a lower gain threshold is expected compared to an optimized single nanowire monomer. With the uncoated nanowires, the smallest modal cost function value is obtained with the tetramer T${}_{b}$ mode based on hybridization of TE${}_{01}$ modes in the nanowires (Table 1). Furthermore, both the HE${}_{11}$-based oligomer modes D${}_{a}$ and T${}_{a}$ and the TE${}_{01}$-based oligomer modes D${}_{b}$ and T${}_{b}$ offer a clear reduction in the minimum modal cost function value compared to the corresponding monomer modes. The improvements essentially arise from increased modal confinement factor and increased substrate end modal reflection coefficient magnitude. However, the main limiting factor for all these modes seems to be a trade-off between the substrate and superstrate end modal reflection coefficient magnitude which have opposite trends in their geometry (diameter and separation of the nanowires) dependence. With the coated nanowires, the smallest modal cost function value is obtained with the tetramer T${}_{\mathrm{ca}}$ mode based on hybridization of HE${}_{11}$ modes in the nanowires (Table 2). Interestingly, the HE${}_{11}$-based coated oligomer modes D${}_{\mathrm{cb}}$ and T${}_{\mathrm{ca}}$ with TE${}_{01}$-like transverse fields can offer a clear reduction in the minimum modal cost function value compared to the corresponding coated monomer modes, while the other (in a sense, oppositely arranged) HE${}_{11}$-based coated oligomer modes D${}_{\mathrm{ca}}$ and T${}_{\mathrm{cb}}$ can offer only modest improvement at best. The coated oligomer modal fields concentrated in the coating do not experience a similar trade-off in substrate and superstrate modal reflection coefficient magnitude as with the uncoated oligomers, but the maximum modal confinement factor and modal reflection coefficient magnitude values reached are all smaller leading to overall larger modal cost function values (i.e., larger expected threshold gain). Note that, despite the observed further threshold gain reduction with the tetramers compared to the dimers, this benefit is rather small in comparison to the increased footprint and volume (especially with the uncoated nanowires).

It is also insightful to compare these results with those reported for other nanowire laser structures. For example, in Ref. [4], a cylindrical GaAs nanowire (n=3.63 at 870 nm wavelength and room temperature) with around 360 nm diameter lying horizontally on a SiO${}_{2}$ substrate was simulated to yield (bulk material) threshold gain of approximately 300 cm${}^{-1}$ with the TE${}_{01}$ mode in a 6 μm long nanowire (when approximating the propagation losses as much smaller than the mirror losses, similar as in the present study in Equation (2)). With our uncoated dimer D${}_{b}$ mode having minimum modal cost function value of 0.913, the (bulk material) threshold gain for L=6 μm would be approximately 1520 cm${}^{-1}$ (with equation 2). This is roughly five times higher than in the horizontal nanowire above despite approximately twice the active region volume (although a quantitative comparison is strictly valid only at the same wavelength). Additionally, contrary to our case of vertical nanowires, the horizontally lying nanowire had the benefit of large modal reflectivity at both ends. On the other hand, to the best of our knowledge, the Er-doped alumina has so-far not yet been employed in laser structures. However, in Ref. [13], it was used to make an on-chip optical amplifier with a hybrid slot waveguide structure (Er-doped alumina filling the slot between Si${}_{3}$N${}_{4}$ strips on SiO${}_{2}$). The modal confinement factor of the most confined mode in the 100 nm wide and 460 nm tall slot was estimated via simulation as 0.315, which is significantly less than in our coated nanowire waveguides (Table 2, note that we use the same wavelength and Er-doped alumina refractive index in our study). However, although the Er-doped alumina gain is high for a coating material, it might still not be sufficient, and even better modal properties would be required for a mode to reach lasing. Therefore, with the present results, the uncoated nanowire oligomer lasers would seem to be the more promising approach.

Since the uncoated nanowire oligomer modes, while otherwise promising, don’t necessarily yield high enough modal reflection coefficient magnitude at the substrate end, it could be worthwhile to consider additional reflective structures there. One option would be to consider classical planar distributed Bragg reflector stacks on the substrate, although these might not be as easily realized on the (111) plane substrates needed for vertical bottom-up nanowire growth. Another interesting option then would be to use narrow nanowire bottom-up growth through a thick SiO${}_{2}$ mask layer and lateral overgrowth to reach the proper nanowire waveguide diameter above the mask. This approach was demonstrated in Ref. [6], where surface passivated GaAs/AlGaAs/GaAs core–shell nanowire lasers with such mask layer on top of a Si substrate were estimated (via simulations) to have up to 40% modal reflectivity for the HE${}_{11}$ mode (corresponding to modal reflection coefficient magnitude of approximately 0.63) compared to below 1% when grown directly on the Si substrate (this was achieved with nanowire diameter of 80 nm and 470 nm inside and above the 160 nm thick mask layer, respectively). Even higher modal reflectivities were estimated for higher order modes. On the other hand, with much thicker nanowires, strongly confined helically propagating modes would become supported, as reported in Ref. [23], where lasing was demonstrated for InGaAs nanowires with 540 nm to 680 nm diameter and composition for lasing wavelength in the range of 890 nm to 930 nm. The essential feature of these modes is extremely small leakage to the substrate even with very low refractive index contrast between the nanowire and substrate (approximately 0.1 in their study). However, these cavity modes are not of Fabry–Pérot type, which prevents direct comparison with our results. Furthermore, it has been argued [6] that such thick-nanowire modes lead to complex far-field emission patterns and low spontaneous emission coupling to the lasing mode. These could actually be valid concerns for our oligomer modes as well.

Indeed, further study on the topic is clearly called for. First, with increased computational budget, the parameter space of this study could be expanded and higher order monomer modes and other oligomers could also be considered. It could also be worthwhile to consider nanowires with cross-sections of lower symmetry [17]. Second, the above mentioned (or other) structures for enhanced substrate end modal reflection with bottom-up grown uncoated nanowires could be investigated. Third, a more thorough model including possible surface passivation layers, optical pumping excitation, gain modeling via rate equations, and emission extraction to far-field or nearby waveguides would be needed to better assess actual laser performance. The modeling results, whether from a complex or simplified model, could also be verified against measurements conducted on actual fabricated samples. Finally, single-mode lasing would be the preferred operation mode, and it would hence be of interest to investigate schemes to promote and achieve this. Such schemes could include self-selection mechanism via gain region positioning [24] and the Vernier effect via evanescent coupling of modes in nanowires of dissimilar cross-sections [25].

## Figures and Tables

**Table 1:** Minimum modal cost function ($f_{\mathrm{mc}}$, Equation (3)) values in the studied parameter space with the selected uncoated nanowire oligomer modes. The corresponding modal confinement factors (Γ), substrate, and superstrate modal reflection coefficient magnitudes ($\left|r_{\mathrm{sub}}\right|$ and $\left|r_{\sup}\right|$), diameter of the nanowires ($d_{\mathrm{nw}}$), and separation of the nanowires (*s*) are also listed.

Mode	$f_{\mathrm{mc}}$	Γ	$\left|r_{\mathrm{sub}}\right|$	$\left|r_{\sup}\right|$	$d_{\mathrm{nw}}$ [nm]	*s* [nm]
Monomer HE${}_{11}$	1.956	1.416	0.158	0.396	330	-
Monomer TE${}_{01}$	1.105	1.265	0.352	0.703	395	-
Monomer TM${}_{01}$	3.320	0.501	0.397	0.476	500	-
Dimer D${}_{a}$	1.208	1.798	0.269	0.424	345	10
Dimer D${}_{b}$	0.913	1.349	0.399	0.732	375	100
Tetramer T${}_{a}$	1.246	1.779	0.278	0.393	330	80
Tetramer T${}_{b}$	0.912	1.411	0.349	0.791	400	400

**Table 2:** Minimum modal cost function ($f_{\mathrm{mc}}$, Equation (3)) values in the studied parameter space with the selected coated nanowire oligomer modes. The corresponding modal confinement factors (Γ), substrate, and superstrate modal reflection coefficient magnitudes ($\left|r_{\mathrm{sub}}\right|$ and $\left|r_{\sup}\right|$), diameters of the nanowires ($d_{\mathrm{nw}}$), coating thicknesses ($t_{c}$), and separations of the nanowires (*s*) are also listed.

Mode	$f_{\mathrm{mc}}$	Γ	$\left|r_{\mathrm{sub}}\right|$	$\left|r_{\sup}\right|$	$d_{\mathrm{nw}}$ [nm]	$t_{c}$ [nm]	*s* [nm]
Coated monomer HE${}_{11}$	2.574	1.107	0.286	0.202	200	400	-
Coated monomer TE${}_{01}$	2.619	0.968	0.258	0.307	280	400	-
Coated monomer TM${}_{01}$	3.054	0.794	0.301	0.294	300	400	-
Coated dimer D${}_{\mathrm{ca}}$	2.496	1.084	0.290	0.231	200	400	400
Coated dimer D${}_{\mathrm{cb}}$	1.896	1.249	0.251	0.373	260	220	120
Coated tetramer T${}_{\mathrm{ca}}$	1.607	1.177	0.315	0.479	220	200	80
Coated tetramer T${}_{\mathrm{cb}}$	2.204	1.259	0.167	0.372	260	200	10

## Appendix A

Here, we briefly present the field overlap integral equations used for obtaining the propagating power of a specific mode in a 3D full-field waveguide solution. The waveguide can be a single nanowire monomer or any nanowire oligomer as long as the media involved have negligible imaginary part of the refractive indices. A more comprehensive treatment can be found in standard textbooks on guided optics (see, e.g., Refs. [26,27,28]).

The fields of a guided mode ν propagating in the positive *z*-direction can be written as:

(A1) $${\bar{E}}_{\nu }\left(x,y,z\right)=\left({\bar{e}}_{\nu ,t}\left(x,y\right)+e_{\nu ,z}\left(x,y\right){\bar{u}}_{z}\right)e^{i\beta_{\nu }z}$$

and

(A2) $${\bar{H}}_{\nu }\left(x,y,z\right)=\left({\bar{h}}_{\nu ,t}\left(x,y\right)+h_{\nu ,z}\left(x,y\right){\bar{u}}_{z}\right)e^{i\beta_{\nu }z}\;$$

where the transverse components ${\bar{e}}_{\nu ,t}$ and ${\bar{h}}_{\nu ,t}$ are real and the longitudinal components $e_{\nu ,z}$ and $h_{\nu ,z}$ are purely imaginary. Likewise, the guided mode −ν fields propagating in the negative *z*-direction can be written as:

(A3) $${\bar{E}}_{-\nu }\left(x,y,z\right)=\left({\bar{e}}_{\nu ,t}\left(x,y\right)-e_{\nu ,z}\left(x,y\right){\bar{u}}_{z}\right)e^{-i\beta_{\nu }z}$$

and

(A4) $${\bar{H}}_{-\nu }\left(x,y,z\right)=\left(-{\bar{h}}_{\nu ,t}\left(x,y\right)+h_{\nu ,z}\left(x,y\right){\bar{u}}_{z}\right)e^{-i\beta_{\nu }z}\;.$$

The power carried by these guided modes ν and −ν in the positive *z*-direction can be obtained with the Poynting vector as

(A5) $$\gamma_{\nu }=\int_{S_{\infty }}{\bar{S}}_{\nu }\cdot {\bar{u}}_{z}dxdy=\frac{1}{4}\int_{S_{\infty }}\left\{{\bar{E}}_{\nu }\times {\bar{H}}_{\nu }^{*}+{\bar{E}}_{\nu }^{*}\times {\bar{H}}_{\nu }\right\}\cdot {\bar{u}}_{z}dxdy$$

and

(A6) $$\gamma_{-\nu }=\int_{S_{\infty }}{\bar{S}}_{-\nu }\cdot {\bar{u}}_{z}dxdy=-\int_{S_{\infty }}{\bar{S}}_{\nu }\cdot {\bar{u}}_{z}dxdy=-\gamma_{\nu }\;$$

respectively, where $S_{\infty }$ denotes the entire *xy*-plane and ${}^{*}$ denotes the complex conjugate.

In general, there can simultaneously be multiple guided modes due to which the following orthogonality relation is needed:

(A7) $$\int_{S_{\infty }}\left\{{\bar{e}}_{\nu }\times {\bar{h}}_{\xi }^{*}+{\bar{e}}_{\xi }^{*}\times {\bar{h}}_{\nu }\right\}\cdot {\bar{u}}_{z}dxdy=0\;$$

for all modes ν≠ξ. Equation (A7) also holds for ξ=−ν without any additional modifications, which is important, as we specifically need to distinguish between forward and backwards propagation of the same mode. The mode orthogonality also holds for leaky and radiation modes, which essentially means that a guided mode propagating along a translationally invariant waveguide does not exchange energy with any other modes.

With the orthogonality relation, the power carried in the positive *z*-direction by a specific mode component of the total field can be conveniently obtained as

(A8) $$P_{\nu }={\left|\frac{1}{4}\int_{S_{\infty }}\left\{\bar{E}\times {\hat{\bar{H}}}_{\nu }^{*}+{\hat{\bar{E}}}_{\nu }^{*}\times \bar{H}\right\}\cdot {\bar{u}}_{z}dxdy\right|}^{2}$$

or

(A9) $$P_{-\nu }=-{\left|\frac{1}{4}\int_{S_{\infty }}\left\{\bar{E}\times {\hat{\bar{H}}}_{-\nu }^{*}+{\hat{\bar{E}}}_{-\nu }^{*}\times \bar{H}\right\}\cdot {\bar{u}}_{z}dxdy\right|}^{2}$$

using the normalized modal fields

(A10) $${\hat{\bar{E}}}_{\pm \nu }\left(x,y,z\right)=\frac{1}{\sqrt{\gamma_{\nu }}}{\bar{E}}_{\pm \nu }\left(x,y,z\right)$$

and

(A11) $${\hat{\bar{H}}}_{\pm \nu }\left(x,y,z\right)=\frac{1}{\sqrt{\gamma_{\nu }}}{\bar{H}}_{\pm \nu }\left(x,y,z\right)\;.$$

Furthermore, in order to facilitate numerical evaluation, Equations (A5), (A8), and (A9) can be written as

(A12) $$\gamma_{\nu }=\frac{1}{2}\int_{S_{\infty }}\left\{E_{\nu x}H_{\nu y}^{*}-E_{\nu y}H_{\nu x}^{*}\right\}dxdy\;$$

(A13) $$P_{\nu }=\frac{1}{16\gamma_{\nu }}{\left|\int_{S_{\infty }}\left\{E_{x}H_{\nu y}^{*}-E_{y}H_{\nu x}^{*}+E_{\nu x}^{*}H_{y}-E_{\nu y}^{*}H_{x}\right\}dxdy\right|}^{2}\;$$

and

(A14) $$P_{-\nu }=-\frac{1}{16\gamma_{\nu }}{\left|\int_{S_{\infty }}\left\{-E_{x}H_{\nu y}^{*}+E_{y}H_{\nu x}^{*}+E_{\nu x}^{*}H_{y}-E_{\nu y}^{*}H_{x}\right\}dxdy\right|}^{2}\;$$

respectively.
